# Greenhouse Gas and Air Pollutant Emissions from Composting

**DOI:** 10.1021/acs.est.2c05846

**Published:** 2023-01-31

**Authors:** Sarah L. Nordahl, Chelsea V. Preble, Thomas W. Kirchstetter, Corinne D. Scown

**Affiliations:** † Energy Technologies Area, Lawrence Berkeley National Laboratory, 1 Cyclotron Road, Berkeley, California 94720, United States; ‡ Department of Civil and Environmental Engineering, University of California, Berkeley, Berkeley, California 94720, United States; § Biosciences Area, Lawrence Berkeley National Laboratory, 1 Cyclotron Road, Berkeley, California 94720, United States; ∥ Joint BioEnergy Institute, 5885 Hollis Street, Emeryville, California 94608, United States; ⊥ Energy & Biosciences Institute, University of California, Berkeley, Berkeley, California 94720, United States

**Keywords:** Greenhouse Gases, Composting, Air Quality, Ammonia, Methane, Anaerobic Digestion

## Abstract

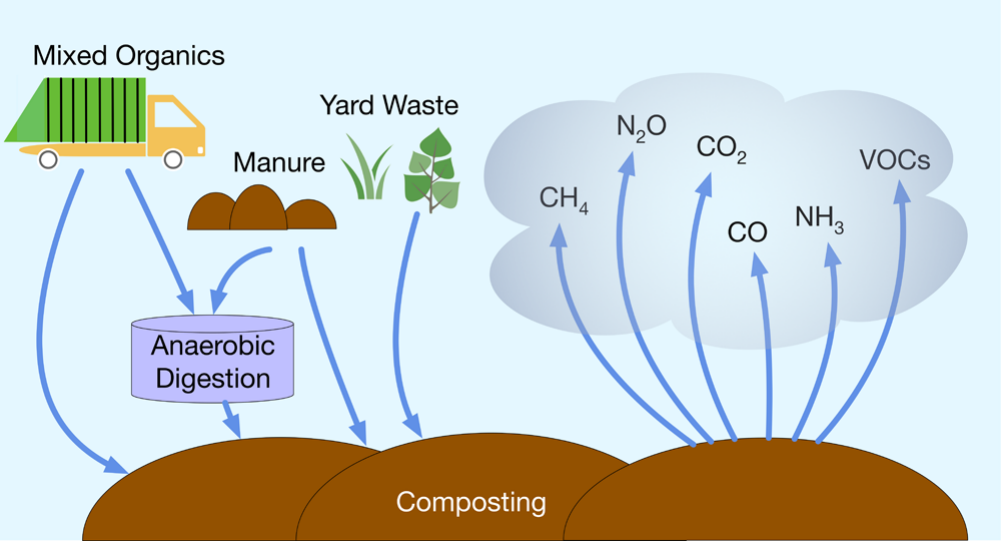

Composting can divert organic waste from landfills, reduce landfill methane emissions, and recycle nutrients back to soils. However, the composting process is also a source of greenhouse gas and air pollutant emissions. Researchers, regulators, and policy decision-makers all rely on emissions estimates to develop local emissions inventories and weigh competing waste diversion options, yet reported emission factors are difficult to interpret and highly variable. This review explores the impacts of waste characteristics, pretreatment processes, and composting conditions on CO_2_, CH_4_, N_2_O, NH_3_, and VOC emissions by critically reviewing and analyzing 388 emission factors from 46 studies. The values reported to date suggest that CH_4_ is the single largest contributor to 100-year global warming potential (GWP_100_) for yard waste composting, comprising approximately 80% of the total GWP_100_. For nitrogen-rich wastes including manure, mixed municipal organic waste, and wastewater treatment sludge, N_2_O is the largest contributor to GWP_100_, accounting for half to as much as 90% of the total GWP_100_. If waste is anaerobically digested prior to composting, N_2_O, NH_3_, and VOC emissions tend to decrease relative to composting the untreated waste. Effective pile management and aeration are key to minimizing CH_4_ emissions. However, forced aeration can increase NH_3_ emissions in some cases.

## 1 Importance of Composting Emissions

Composting is an essential part of any strategy to divert organic waste and reduce fugitive methane (CH_4_) emissions from landfills.^1,2^ In the United States (U.S.), 6–9% of total municipal solid waste is currently composted, although as much as 34% could be composted if all food and yard waste were diverted from landfills.^3,4^ Composting can treat organic waste directly or treat solids remaining after organic waste has undergone anaerobic digestion (AD), ultimately reducing the total mass of waste through aerobic biochemical decomposition and yielding soil amendments for agricultural or landscaping applications. Most reported values for mass loss during composting on a dry basis fall in the range of 10–60%.^5−8^ The motivations for composting are (1) avoidance of fugitive CH_4_ emissions associated with the anaerobic decomposition that occurs in solid waste landfills and manure storage lagoons, (2) the diversion of organic waste from landfills, and (3) generation of compost that is free of harmful pathogens and ready for use in agricultural applications or for erosion control.^9,10^ Although avoiding CH_4_ emissions from landfills is one of the motivations for composting organic waste, the composting process itself emits greenhouse gases (GHGs) and air pollutants, and these emissions are still not well understood.^11^

Gaseous emissions from the composting of organic waste have impacts on both climate change and air quality. The GHG emissions are directly relevant to policy. For example, life-cycle GHG emissions from bioenergy production routes—some of which incorporate composting of residual solids—must be thoroughly documented in the U.S., as they are tied to the Renewable Fuel Standard Renewable Identification Numbers (RINs) and California’s Low Carbon Fuel Standard (LCFS) Carbon Intensity (CI) scores, both of which carry substantial monetary value.^12^

Non-GHG air pollutant emissions from composting facilities affect local and regional air quality and, as a result, human health in surrounding communities.^13^ Emissions of ammonia (NH_3_) and volatile organic compounds (VOCs) from composting are of particular concern because they are precursors of secondary fine particulate matter (PM_2.5_), which is the primary driver of air pollution-related health impacts.^14,15^ A detailed description of NH_3_ emissions and PM_2.5_ formation is provided in the Supporting Information. VOCs are also precursors to tropospheric ozone formation which impacts human health and sensitive vegetation and ecosystems.^14^ Lastly, VOCs and NH_3_ have low odor detection thresholds and can cause a public nuisance for surrounding communities. Odorous pollutants can impact permitting for new facilities, particularly in nonattainment areas in the U.S. where ambient air pollutant concentrations exceed the National Ambient Air Quality Standards. Our review covers NH_3_ and VOC emissions but does not include other odorous compounds such as noncarbon-containing volatile sulfur compounds (e.g., hydrogen sulfide). Because of the additional environmental and human health impacts from non-GHG emissions, it is essential to balance ambitious landfill diversion goals with local air quality and odor concerns associated with operating composting facilities.^16^

Despite the importance of GHG and air pollutant emissions from composting, available data can be difficult to interpret and use for policy implementation. The California Air Resources Board released a recommended methodology for estimating composting emissions in 2015, but the method was only applicable to mixtures with at least 85% green waste and a maximum of 15% food waste, biosolids, or manure.^17^ The degree to which specific composting practices and incoming waste composition affect emissions per unit of composted material is not well understood. Furthermore, researchers incorporating composting emissions into life-cycle assessments (LCAs) are often not experts in different measurement techniques and the degree to which measurement methods affect the accuracy of empirical data. This knowledge gap makes prioritization of emissions mitigation strategies and scenario planning for zero-waste policies challenging.

The purpose of this review is to evaluate data available in the scientific literature on air emissions from composting operations, discuss the merits and trade-offs of measurement strategies employed in past studies, and provide guidance for researchers and decision-makers who seek to integrate composting emission factors into policy and environmental impact studies.

Regarding GHGs, we mainly focus on CH_4_ and nitrous oxide (N_2_O) emissions because they are the primary drivers of net climate forcing impacts from composting.^11,18^ Although contemporary carbon emitted as CO_2_ during composting is not thought to have a net climate impact, we also include limited data on CO_2_ emissions results, reported separately from CH_4_ and N_2_O, in the Supporting Information (Figure S1). This review also includes data on NH_3_ and VOCs because of their importance for air quality and air pollution-related human health impacts.^15^ Empirical emission values collected from the literature were differentiated based on the type of material being composted, measurement methods used in the study, and the management strategies employed during the composting process, with the goal of developing more representative and material-specific recommendations for composting emission factor ranges.

Prior reviews have explored some dimensions of this topic but fall short of providing recommended ranges for emission factors that can be used in future LCAs and policy-making. For example, Amlinger et al. (2008) primarily focused on their own measured results for CH_4_, N_2_O, and NH_3_ but included a review of prior results to inform the development of a helpful, mostly qualitative table summarizing the effects of different compost management strategies on emissions and the mechanisms behind those effects.^19^ Brown et al. (2008) reviewed a broader set of literature values on GHG emissions associated with different alternatives for disposing of/treating organic waste, including landfilling and anaerobic digestion, in comparison to composting.^20^ Lou and Nair (2009) compared GHG emissions from composting and landfilling organic waste, concluding that landfilling results in higher GHG emissions as compared to composting, a conclusion that reflects broad consensus in the research community.^21^ Pardo et al. (2015) conducted a meta analysis of 50 studies to establish the relative impacts of different management strategies, such as forced aeration versus turning and the addition of bulking agents.^22^ Pardo et al. (2015) considered the same raw feedstocks as those included in this review and focused on the relative impact of different operational practices and conditions but did not establish emission factors per tonne of waste composted. Bong et al. (2017) and Sayara and Sánchez (2021) provide more qualitative reviews of composting GHG emissions and discuss GHG mitigation strategies.^23,24^ Sayara and Sánchez (2021) summarize research regarding the impact of composting practices and feedstock characteristics on emissions, while Bong et al. (2017) focus more specifically on the variability of scope definition and inventory analysis in published LCAs of composting. Neither review provides recommended emission factors.

Although this review focuses on gaseous emissions during the composting process itself, excluding truck transport and combustion of fuels to operate equipment, composting emission factors are more meaningful in a broader context, where each end-to-end process for managing organic waste can be compared. There are two main competing routes of relevance: (1) composting followed by land application of finished compost and (2) landfilling untreated organic waste. The use phase for finished compost is essential to include in life-cycle emissions inventories; applying compost can reduce the need for synthetic fertilizers and, in some cases, increase the net primary productivity on degraded lands.^25^ The comparison between composting emissions and landfilling organic waste is another important topic, and this has been explored more thoroughly in prior reviews, although gaps in empirical data remain.^21,26^ In the Supporting Information, we provide an overview of the state of knowledge related to how compost application and landfilling organic waste affect net GHG emissions.

## 2 The Role of Composting in Organic Waste Management

Organic wastes that can be composted include the entire organic fraction of municipal solid waste (OFMSW, which includes a variety of organic waste types), food waste, yard waste, sewage sludge, manure, and digestates (residual solids remaining after AD). The most commonly composted material is source-separated yard waste. According to the U.S. Environmental Protection Agency (EPA), approximately 0.4% of food waste and 63% of yard waste are currently composted in the U.S. While technically compostable, paper waste is more commonly recycled unless it is soiled or otherwise unsuitable,^27^ so we have excluded it from this review. Solid digestate can be directly applied to agricultural land as a fertilizer amendment, but there are typically seasonal limitations on this practice due to nutrient runoff concerns in some states, so AD facilities may send digestate to composting facilities during part or all of the year.^15,28−31^

The wastes processed at composting facilities vary in moisture content, the carbon-to-nitrogen ratio (C:N), pH, volatile solids (VS) content, and other characteristics that lead to varying rates of aerobic decomposition and emissions to the atmosphere. VS refers to the part of compostable materials that is combusted at 550 °C in the presence of air after 2 h and can be a proxy for the fraction of biodegradable material.^32^ The composting process itself involves a diverse microbial community, in which the relative abundance and activity level of different microbes shift over time. Because levels of aeration and the composition of organic matter will vary, there is also heterogeneity across a given pile or windrow. The multistage composting process begins with the mesophilic phase, in which mesophilic microbes break down easily degradable compounds until the generated waste heat increases the temperature to 40 °C, which inhibits their growth.^33^ It is during the mesophilic phase that nitrifying and denitrifying bacteria produce N_2_O.^33^ Above 40 °C, the thermophilic microbes begin to dominate, and the increased activity of methanogens results in greater CH_4_ emissions.^33^ At this point, reaching temperatures above 55 °C is desirable because this kills most human and plant pathogens; however, aeration is necessary to prevent the pile from exceeding 65 °C, the threshold where most microbes are killed and the rate of decomposition decreases.^34^ After the thermophilic phase, the compost cools and undergoes a curing and maturation stage, during which slow decomposition continues as mesophilic microbes become dominant again.

Composting operations are designed to facilitate this natural process, and practices at different facilities are distinguished by the manner in which material is stored and aerated, either in windrows or vessels and with manual, passive, or forced aeration. With in-vessel composting, material is contained in a series of containers or concrete bunkers, in which the temperature and air flow are controlled. This approach requires less land area than windrow composting and can be more efficient with proper management but is a more expensive method.^35^ In industrial-scale windrow composting operations, material is placed in rows of long and narrow piles called windrows. These windrows can either be left uncovered or can be enclosed by plastic sheeting or within bags. The dimensions of these piles are typically 2–6 m wide and 1–3 m in height, which is large enough to maintain thermophilic composting conditions while also ensuring adequate aeration.^36,37^ There are several methods of aeration used to ensure the aerobic conditions required for composting. One method is to periodically turn uncovered compost piles manually or mechanically. Alternatively, static piles, either uncovered or enclosed by plastic sheeting, are aerated by natural, passive, or forced means. Natural aeration strictly relies on diffusion for air flow through the pile, but this approach can be inhibited by high moisture content material that reduces air space and increases the likelihood of conditions in the pile becoming anaerobic.^38^ Passively aerated piles include perforated pipes to promote air circulation that is driven by thermal gradients. Forced aeration similarly uses perforated pipes but includes a positively or negatively pressurized pump to either push or pull air through the composting pile on prescribed cycles to control temperature and optimize the composting process. In negative aeration, the air drawn from piles may be treated with a biofilter to control odor and VOCs.^39,40^ Naturally and passively aerated piles compost at a slower rate, whereas the controlled forced aeration or turning of piles results in shorter composting cycles.^41^

## 3 Understanding and Measuring Emissions from Composting

### 3.1 Overview of Key Emission Sources

By mass, CO_2_ is the dominant compound emitted to the atmosphere during composting operations.^33^ During each stage of composting, some carbon present in the organic material is oxidized to CO_2_. Because this CO_2_ production is a natural part of organic decomposition and the carbon present in most compost feedstocks is biogenic (part of the contemporary carbon cycle, in contrast to fossil carbon), these emissions are considered to be climate-neutral.^33,42,43^ Emissions of other air quality and climate-relevant pollutants vary, depending on factors like oxygen availability, temperature, and moisture content. Under anaerobic conditions, decomposition occurs more slowly; and methanogens create greater quantities of CH_4_, while emitted CO_2_ decreases. Localized areas of anaerobic decomposition in composting operations are inevitable, but turning and aeration can minimize CH_4_ emissions. Methanotrophs play an important role in consuming CH_4_ that may be produced in localized anaerobic regions of the pile or windrow; one study suggested that 46–98% of CH_4_ produced during composting operations is consumed by methanotrophs before it can escape to the atmosphere.^44^ Carbon monoxide (CO) formation is well documented, but the mechanisms are still not fully understood by the scientific community.^45^ CO in composting environments can be formed through thermochemical processes, stimulated by heat and ultraviolet radiation, and CO can also be produced and consumed by microbes.^45−47^

Nitrogen cycling in composting operations involves numerous direct and indirect processes, but an understanding of the basic mechanisms is important, given the relevance of N_2_O and NH_3_ emissions to the climate and human health. Biological removal of nitrogen involves nitrification and denitrification and ultimately results in N_2_O emissions.^48^ NH_3_ is produced as microbes consume peptides and amino acids present in protein-rich waste. Nitrification is a two-step process in which microbes oxidize NH_3_ to nitrite (NO_2_^–^) and subsequently oxidize NO_2_^–^ to nitrate (NO_3_^–^). A fraction of the NO_2_^–^ formed will be converted to nitric oxide (NO) and eventually N_2_O by ammonia oxidizing bacteria, rather than forming NO_3_^–^. During denitrification, microbes anaerobically convert NO_3_^–^ back to NO_2_^–^, then to NO, and ultimately to N_2_O, most (but not all) of which is ultimately converted to nitrogen gas (N_2_). NH_3_ can also be directly emitted to the atmosphere, particularly from well-aerated piles where it escapes before microbes are able to oxidize it. NH_3_ emissions from compost increase with increasing aeration, lower C:N ratios, higher temperatures, and higher pH.^49^ The conditions for reducing NH_3_ volatilization, unfortunately, can be counter to the optimal microbial conditions for fast and efficient composting.^50^

### 3.2 Emissions Measurement Methods

Many methods are used to measure emission rates from composting, and each has advantages and disadvantages to consider when interpreting and using the empirical data. Emissions can be characterized in controlled laboratory experiments or with in situ field measurements. Sampling can be conducted continuously in the field with pollutant analyzers or intermittently by collecting discrete samples of emitted gas into canisters or bags that are later analyzed in the laboratory. Pollutant concentrations can be measured at a single point or integrated across the composting pile. The trade-off in temporal and spatial resolution between these sampling approaches depends on the sampling conditions and objectives of the study.

Laboratory experiments have been used to approximate the composting process under controlled conditions in reactors that are typically ∼10–200 L in volume.^51−64^ Lab experiments allow for a better understanding and characterization of how specific environmental conditions, such as temperature, pH, moisture content, and material, affect pollutant emissions than can often be attained with field measurements. However, the smaller lab-scale and experimental conditions may not be representative of the real-world. These emission factors should be used with caution or ideally validated against field measurements for similar materials and conditions.

Measurements can be made in the field as relatively controlled experiments of pilot- or full-scale test windrows that are maintained separately from normal operations.^19,42,65−70^ In situ sampling of full-scale commercial windrows operating under normal composting conditions is also common.^11,71−75^ Ideally, field measurements of emissions would be fully integrated over the windrow or pile surface, over the full duration of the composting cycle, and without disrupting normal composting conditions. This ideal measurement approach is not practical under many sampling scenarios, however, given researcher resources and environmental/operational conditions. As such, many sampling methods have been used for field measurements, including but not limited to flux chambers, gas probes, wind tunnels, open emission chambers, tracer releases, inverse dispersion analysis, micrometeorological mass balance, and high-density spot sampling. Each approach has its limitations, as described below. These emission measurements can also be accompanied by intermittent measurements and/or continuous monitoring of conditions in the windrow, which is important for developing a deeper understanding of the mechanisms driving emissions over time and space. Detailed descriptions of each measurement method and their impacts on reported emission factors are included in the Supporting Information.

### 3.3 Characterization of Composting Emissions Studies in the Current Literature

We conducted a survey of peer-reviewed studies that report CO_2_, CH_4_, N_2_O, NH_3_, and/or VOC emissions from composting, keeping track of feedstock type and composting conditions. Where possible, we converted reported emission factors to units of kilograms of pollutant emitted per kilogram of wet (sometimes referred to as fresh or green) feedstock material composted. Composting emission factors are most commonly reported in terms of wet material because this is practical for commercial operations and general material flow tracking. However, these values should be converted to a per-dry-mass basis for use in carbon or nitrogen balance modeling since water makes up a significant portion of composting feedstocks (36–85%, Table S1). We do not provide emission factors on a dry basis because several studies did not provide sufficient data on moisture content to calculate these conversions.

Studies that did not give enough information to calculate reasonable emission factors and secondary sources that did not provide original data were excluded from our review. In one case, we excluded 6 measured emission factors from further analysis because the authors acknowledged that two of their small-scale measurement methods, a static flux chamber method and a funnel method, significantly underestimated GHG emissions.^76^ In total, 388 emission factors from 46 studies reporting emission measurements were considered in the survey, corresponding to 140 composting scenarios (Table S1).

**Figure 1:**
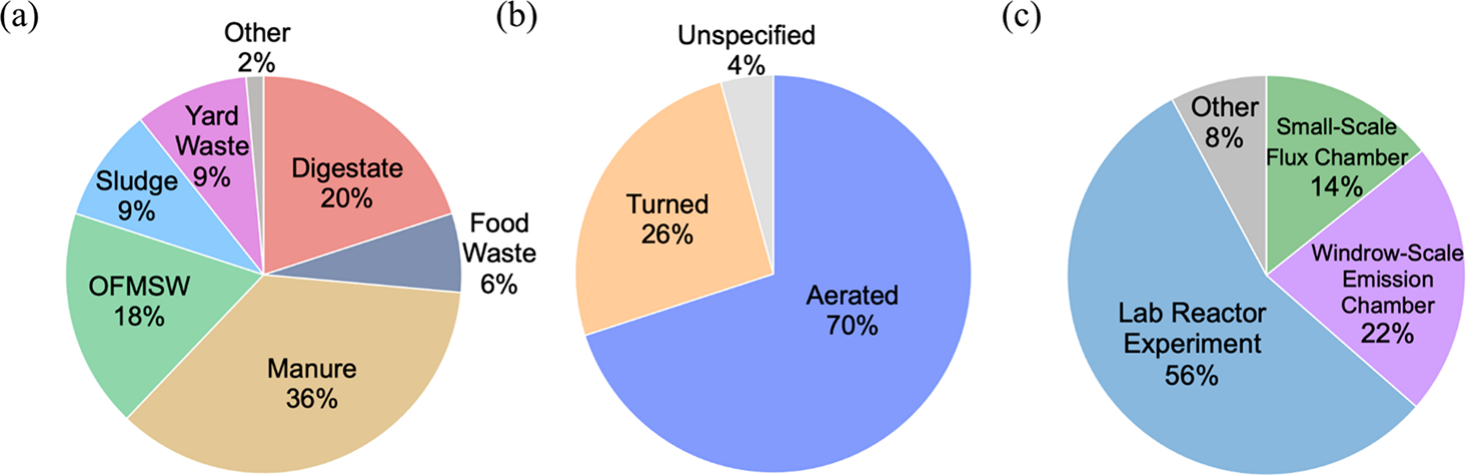
Breakdown of the 140 composting scenarios and study methods associated with the reported emission factors collected for analysis. Emission factors are categorized by (a) composted material, (b) aeration method, and (c) measurement method.

A majority of currently available research on composting emissions is focused on manure composting. Therefore, manure composting comprises most of the emissions observations across all pollutant types (Figure 1 and Table S1). The literature survey does include an extensive accounting of available literature on GHG composting emissions from food waste, OFMSW, yard waste, and anaerobically digested materials. Emissions from the composting of solid digestate are particularly understudied, and given the importance of these emissions for regulatory decision-making in waste-to-energy pathways, this topic requires further research. Of the collected data, most emission factors are associated with forced aeration (Figure 1). This is not necessarily the most common industry practice but is more easily replicated in lab-based studies (Figure 1). Most commercial composting operations involve outdoor windrows that can be turned or forcibly aerated, are not equipped with effective emission control systems, and allow all fugitive emissions to be released to the atmosphere. Alternatively, in-vessel or fully enclosed composting facilities can more easily be equipped with scrubbers and biofilters to reduce atmospheric emissions.^77^

## 4 Composting Emission Factors by Source Material and Management Practices

### 4.1 Greenhouse Gas Emissions

Establishing definitive, broadly applicable GHG emission factors for composting organic wastes is difficult because emissions vary due to a number of factors beyond feedstock (waste) type. These include the following: local climatic conditions at the composting site; composting method and duration; aeration method and frequency; use of a bulking agent intended to provide structure to piles/windrows and facilitate aeration; and the feedstocks’ VS content, C:N ratio, moisture content, and pH. In this section, we differentiate previously published emission factors based on source material and management practices to elucidate the impact of these variables on GHG emissions. Our discussion of GHG emissions from composting is focused on CH_4_ and N_2_O, as these gases are most likely to drive net changes in radiative forcing from composting operations. Biogenic CO_2_, by contrast, is not included in our GHG footprint calculations because it is part of the contemporary carbon cycle and will be resequestered during plant regrowth.^20,33,42,43,78^ However, depending on how a particular researcher or practitioner chooses to account for carbon flows, it may be important to account for CO_2_. Further information and emission factor distributions for CO_2_ are discussed in the Supporting Information.

#### 4.1.1 Variation by Feedstock Type

**Figure 2:**
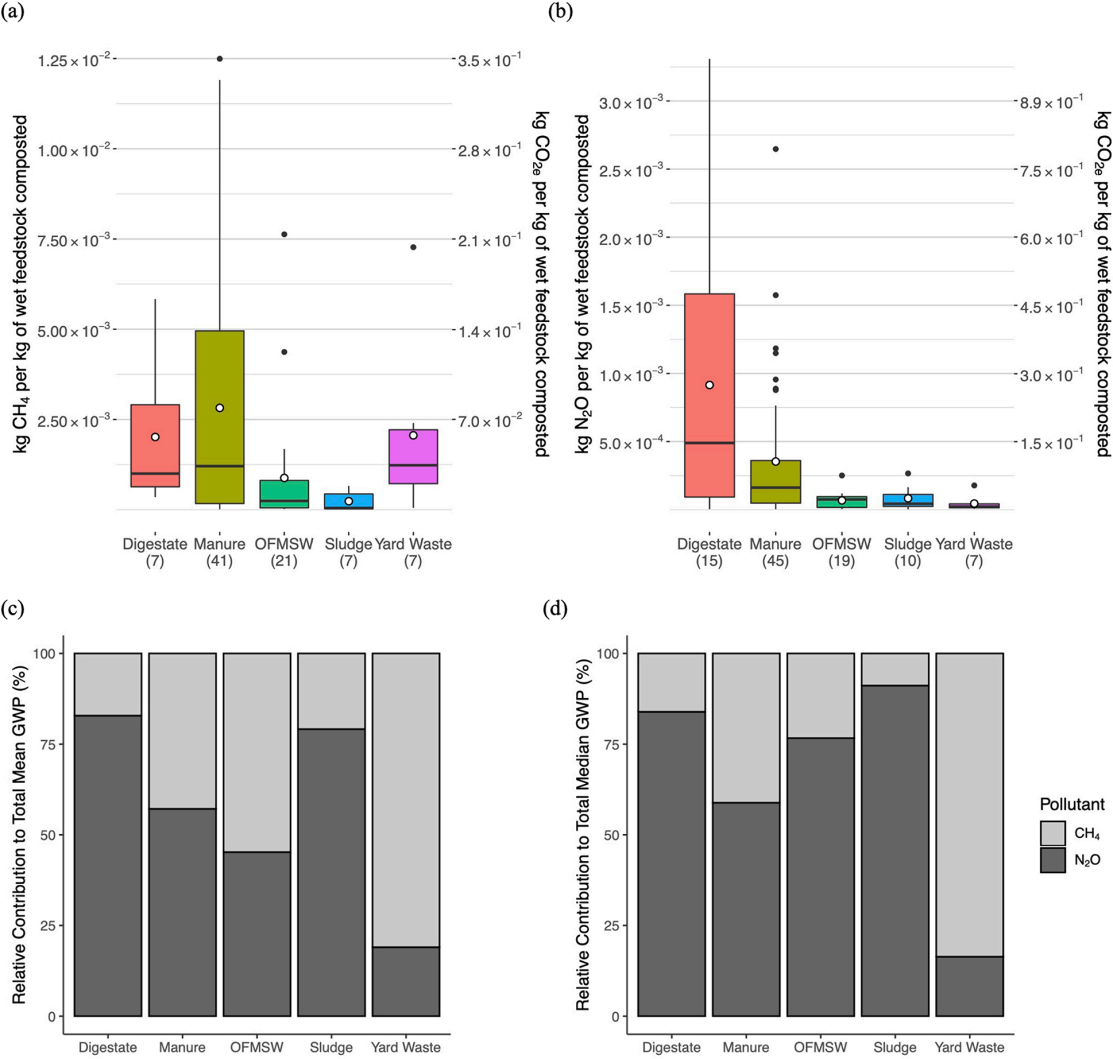
Distributions of (a) CH_4_ and (b) N_2_O emission factors for composting reported in the literature and relative contribution to total GWP_100_ based on (c) mean values and (d) median values. The sample size (*n*) of data points contributing to each boxplot is indicated in the *x*-axis labels for parts (a) and (b); the first value refers to the sample size of CH_4_ emission factors, and the second value refers to that of N_2_O emission factors. Parts (a) and (b) have two *y*-axes: the left axis indicates the per-tonne mass of the specified pollutant emitted (exact values), and the right axis shows the CO_2_-equivalent emission factor (rounded values), so that CH_4_ and N_2_O emissions can be compared with respect to GWP_100_. The mean values for the boxplot data are indicated by the open point symbols, while outliers are shown as closed circles.

GHG emission factors by feedstock type are presented in Figure 2 and Table 1. Figure 2 shows the distributions of CH_4_ and N_2_O emission factors by feedstock type (manure, OFMSW, sludge, and yard waste) and for digestate. The distribution for digestate includes data across all original feedstocks to allow for a general comparison to raw material composting; the effect of AD as a pretreatment to composting is discussed in more detail in Section 4.1.3. We grouped together studies examining OFMSW, household waste, kitchen waste, and food waste because of ambiguous distinctions between these feedstocks. If yard waste is collected separately and paper/paperboard is recycled, the remaining OFMSW will be primarily composed of food, food-soiled paper products, and other paper products that cannot be recycled.^15^ However, in that case, a composter processing this high-moisture food waste-dominated material will likely need to add a bulking agent, such as wood chips, sawdust, dry leaves, shredded paper/cardboard, or other materials that are very similar to yard waste and/or paper and paperboard. Therefore, the final material that is composted in all of these studies is likely to be similar regardless of whether yard waste and/or paper/paperboard in the original waste stream are diverted for other uses.

**Table 1:** Summary of GHG Emission Factor Data for Composting Raw Materials^a^

		emission factor				
		kg pollutant/kg of wet feedstock		kg CO_2e_/kg of wet feedstock		
feedstock	pollutant	mean	median	mean	median	sample size
manure	CH_4_	2.82 × 10^–3^	1.21 × 10^–3^	7.90 × 10^–2^	3.39 × 10^–2^	41
	N_2_O	3.54 × 10^–4^	1.62 × 10^–4^	1.05 × 10^–1^	4.83 × 10^–2^	45
	CO_2_	1.40 × 10^–1^	1.47 × 10^–1^	1.40 × 10^–1^	1.47 × 10^–1^	30
OFMSW	CH_4_	8.79 × 10^–4^	2.43 × 10^–4^	2.46 × 10^–2^	6.80 × 10^–3^	21
	N_2_O	6.80 × 10^–5^	7.50 × 10^–5^	2.03 × 10^–2^	2.24 × 10^–2^	19
	CO_2_	5.63 × 10^–2^	4.30 × 10^–2^	5.63 × 10^–2^	4.30 × 10^–2^	3
sludge	CH_4_	2.34 × 10^–4^	4.50 × 10^–5^	6.55 × 10^–3^	1.26 × 10^–3^	7
	N_2_O	8.36 × 10^–5^	4.36 × 10^–5^	2.49 × 10^–2^	1.3 × 10^–2^	7
	CO_2_	1.75 × 10^–2^	1.75 × 10^–2^	1.75 × 10^–2^	1.75 × 10^–2^	2
yard waste	CH_4_	2.06 × 10^–3^	1.23 × 10^–3^	5.77 × 10^–2^	3.44 × 10^–2^	7
	N_2_O	4.54 × 10^–5^	2.27 × 10^–5^	1.35 × 10^–2^	6.76 × 10^–3^	7
	CO_2_	1.71 × 10^–1^	1.56 × 10^–1^	1.71 × 10^–1^	1.56 × 10^–1^	4

a Digestate is excluded in this table because of variation in the original raw feedstock materials.

Table 1 provides mean and median emission factors by feedstock type, which can be useful for researchers and LCA practitioners who must approximate composting emissions as part of their analyses of waste management or waste-to-energy systems. It is important to note that assembling results from all prior field- or lab-based research may not provide a representative sample of real-world composting operations. For example, the majority of emissions data for composting manure came from studies examining either beef cattle, dairy cattle, or swine manure. In almost all studies considered here, composting operations for yard waste involved open, turned windrows. Most surveyed studies of sludge composting emissions were lab-based, involving closed reactors and forced aeration, and only examined wastewater treatment sludge.

Based on 100-year global warming potential (GWP_100_) values for N_2_O and CH_4_ (GWP_100_ is equal to 298 and 28, respectively.), the median emission values for sludge, digestate, and OFMSW suggest that N_2_O is the largest contributor to total CO_2_-equivalent (CO_2e_) emissions, while CH_4_ emissions are higher on a CO_2e_ basis for yard waste (Figure 2). Manure composting resulted in the highest total GWP_100_, with a roughly even split between CH_4_ and N_2_O on a CO_2e_ basis (Figure 2, Table 1). Pardo et al. (2015) similarly found that composting manure resulted in the highest CH_4_ and N_2_O emissions when comparing across different feedstocks.^22^

#### 4.1.2 Impact of Feedstock Characteristics

In addition to the type of feedstock (e.g., manure, food waste), measurable characteristics including moisture content, VS content, and pH play a role in determining emissions. We attempted linear regressions using the ordinary least-squares method and more robust regressions using M-estimation to assess the relationship between each feedstock characteristic listed in Table S1 (% bulking agent, VS content, C:N ratio, moisture content, and pH) and each emission factor. The observed relationships were not statistically significant, even when controlling for feedstock type or measurement methods. However, a few general trends emerged, consistently offering residual standard error less than 0.01 with varying degrees of freedom. The data collected in the literature survey suggests a positive correlation between moisture content and CH_4_ emissions, and this holds true when controlling for feedstock type, which is supported by results from Pardo et al. (2015).^22^ There is limited data that may suggest a negative correlation between moisture content and N_2_O emissions (*n* = 84) and a positive correlation between VS content and N_2_O emissions (*n* = 22), but further study is required to support any definitive conclusions. The collected data does not support a correlation between the C:N ratio and GHG emissions, and the impact of pH on overall emissions is likely negligible. Contrary to the results of our literature survey and analysis, Jiang et al. (2011) found in a lab-based study of swine manure composting that moisture content did not significantly impact CH_4_ emissions, the C:N ratio was negatively correlated with CH_4_ emissions, and neither moisture content nor the C:N ratio had an impact on N_2_O emissions.^53^

#### 4.1.3 Impact of Anaerobic Digestion Prior to Composting

The literature on GHG emissions from the composting of digestate is limited, making it difficult to draw conclusions about the impact of AD as a strategy for pretreating organic waste prior to composting. However, even with the limited data available, there are some basic relationships that can be used to approximate differences in composting emissions between post-AD material and untreated material.

Li et al. (2018) provide one of the only studies that directly compares emissions from the composting of post-AD digestate to the same undigested material as a control.^63^ In this lab-based experimental study, Li et al. composted raw, untreated feedstock—a mixture of manure and agricultural residues—as well as feedstock that first underwent AD for varied digestion times. Corn stover was added to ensure a similar bulk density across all samples during composting. CH_4_ emissions during composting increased relative to the nondigested control treatment when the feedstock material underwent AD for only 15 days but decreased when the digestion time was 30 or 45 days. Without additional data on the microbiomes and volatile solids content in these composting experiments, it is only possible to speculate as to why shorter AD residence times caused elevated CH_4_ emissions during subsequent composting. It is possible that insufficient residence times during AD may allow digestate to be “seeded” with methanogens.^63^ It is also possible that insufficient AD residence times result in higher concentrations of intermediate products from the hydrolysis, acidogenesis, and acetogenesis stages of AD in the final digestate. Additional studies and data would be required to support the development of feedstock-specific composting emission factors for post-AD materials.

Li et al. (2018) offer more conclusive results regarding the impact of AD on N_2_O emissions from composting, which can be the primary contributor to total GWP_100_ from composting (Figure 2).^63^ Piles pretreated with AD had 57–81% lower N_2_O emissions relative to the nondigested control. Longer digestion times resulted in further reductions in N_2_O emissions. Li et al. reported an average VS reduction during AD of 61% (individual VS reduction data for each batch treated with AD was not reported). For perspective, the mean N_2_O emission factor reported by Li et al. for post-AD manure is 69% lower than the mean N_2_O emission factor for composting untreated manure in Table 1.

In addition to Li et al. (2018) study, seven other studies measured GHG emissions from composting digested materials but did not include controls (identical untreated materials).^11,54,61,62,64,71,77,79^ For instance, Colón et al. (2012) included a comparison of in-vessel composting with and without AD pretreatment, finding that N_2_O emissions were 53% lower and CH_4_ emissions were ∼7 times higher for OFMSW treated with AD relative to raw OFMSW.^77^ These results support the assertion that AD can reduce N_2_O emissions from composting, but it is important to note that Colón et al. observed real-world operations at facilities with similar but not identical OFMSW feedstocks. Maulini-Duran et al. (2013) compared two different types of wastewater treatment sludge: (1) sludge sent directly from a wastewater treatment facility to composting without undergoing AD and (2) sludge at a separate facility, treated with AD and subsequently sent to composting.^54^ They found that CH_4_ and N_2_O emissions were respectively 60 and >100 times higher for the post-AD material. However, there was not a proper control in this study because the source material originated from entirely different facilities. The N_2_O emission factors reported by Maulini-Duran et al. for composting post-AD sludge aligned better with N_2_O emissions reported by several other studies for composting raw, untreated sludge.^19,60^

Preble et al. (2020) measured emissions at a commercial-scale composting facility that processed digestate remaining after dry (high-solids) AD of OFMSW and calculated GHG emission factors per unit of incoming material.^11^ This study did not include a control comparison to untreated OFMSW. However, it is notable that their reported N_2_O emission factor is approximately 80% lower than mean and median N_2_O emission factors for composting untreated OFMSW shown in Table 1. Conversely, Preble et al. report a CH_4_ emission factor that is ∼5 times higher than the mean value and ∼18 times higher than the median value for untreated OFMSW (Table 1). Although Preble et al. did not directly measure VS reduction during the dry AD process, the EPA WARM uses a VS reduction of 75% during AD of municipal food waste, a reasonable proxy for OFMSW.^80^

Like Preble et al. (2020), Beylot et al. (2015) studied the emissions from composting post-AD OFMSW and observed N_2_O emissions that were 75% lower than what is reported by Preble et al. and per-tonne CH_4_ emissions that were ∼30% higher.^79^ Zeng et al. (2016) conducted a series of lab-based trials to assess nitrogen emissions from composting digested OFMSW under a variety of conditions, including varied bulking agents, feedstock mixing ratios, and initial moisture content, and found N_2_O emissions ranging from 5.6 × 10^–4^ to 3.3 × 10^–3^ kg per tonne of wet feedstock.^61^ This range is higher than what has been reported from field measurements of both pre- and post-AD OFMSW composting. While lab-based experiments can be useful for comparing a range of materials and conditions under controlled conditions, we advise against relying on these values to represent commercial composting conditions.

Based on the limited data available on emissions from pre- and post-AD organic waste, the question is whether there is a defensible method for approximating differences in composting emissions in the absence of reliable measured data. The studies reviewed here suggest that treating waste with AD, thereby lowering its VS content, can subsequently reduce N_2_O emissions during composting relative to the alternative approach of sending untreated material straight to composting without AD. For researchers and practitioners who must approximate emission factors for composting digestate, it may be appropriate to select a measured emission factor for composting raw materials and apply a reduction factor equivalent to the estimated VS reduction. For example, by applying the lowest observed reduction in N_2_O emissions (57%) from directly comparable emission measurements in Li et al. (2018) to the mean values in Table 1, we estimate that composting digested OFMSW emits 3.9 × 10^–5^ kg of N_2_O per wet tonne and digested manure emits 2.0 × 10^–4^ kg of N_2_O per wet tonne.^63^ In practice, longer AD residence times and greater reductions in VS may lead to further reductions in N_2_O emissions during composting.

Unfortunately, approximating differences in CH_4_ emissions may be more challenging than estimating N_2_O. Li et al. (2018) found that changes in CH_4_ emissions were dependent on AD residence time, with shorter residence times translating to elevated CH_4_ emissions. The increase in CH_4_ emissions when comparing the data for digested OFMSW from Preble et al. (2020) and Beylot et al. (2015) to mean or median values reported in Table 1 may be driven by management practices and/or the fact that the material was anaerobically digested. Highly degradable feedstocks, like manure, OFMSW, and digestate, can create oxygen-depleted zones in compost piles that are compacted and/or not sufficiently aerated, thereby increasing CH_4_ production.^51,54^ Because of variability in pile management and lack of detailed reporting on these practices, it is likely safest to assume that AD has no effect on CH_4_ emissions during composting, provided AD residence times are not below industry standard practices.

#### 4.1.4 Impact of Composting Methods

**Figure 3:**
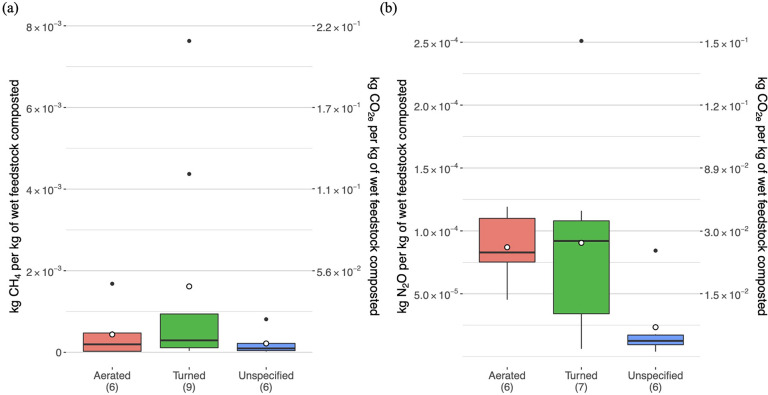
Distributions of (a) CH_4_ and (b) N_2_O emission factors for OFMSW composting based on the aeration method. The sample size (*n*) of data points contributing to each boxplot is indicated in the *x*-axis labels. Each figure has two *y*-axes: the left axis indicates the per-tonne mass of the specified pollutant emitted (exact values), and the right axis shows the CO_2_-equivalent emission factor (rounded values). The mean values for the boxplot data are indicated by the open point symbols, while outliers are shown as closed circles.

As noted previously, different methods for managing compost piles are likely to impact emissions, particularly if some are more effective than others at maintaining aerobic conditions. With regards to composting methods, our analysis focuses on how turning or forced aeration impacts GHG emissions. Using OFMSW as an example, Figure 3 demonstrates the differences in distributions of both CH_4_ and N_2_O emission factors when grouping by the aeration method. The median CH_4_ emission factor was ∼1.5 times higher when the primary aeration method was turning versus forced aeration, and the mean value was nearly 4 times higher. This is supported by a meta-analysis from Pardo et al. (2015), which found turning to be associated with higher GHG emissions.^22^ An important caveat is that this trend may be related to the relatively high number of lab-based studies among those involving forced aeration. Because it is easier to control conditions and maintain proper aeration in laboratory settings that often use enclosed compost reactors, these results may not accurately reflect emissions in industrial scale composting.

As would be expected, several studies have confirmed through measurements that CH_4_ emissions decrease with higher aeration rates, but these studies were less consistent in their findings regarding the impact of aeration on N_2_O emissions.^53,58,60,81^ Unlike the CH_4_ emission factor distributions, the N_2_O emission factor distributions do not diverge significantly based on aeration methods (Figure 3). For instance, the average N_2_O emission factor for composting with turning is only 4% greater than that for composting with forced aeration. There is not sufficient evidence to suggest that the aeration method has a significant impact on N_2_O emissions from composting.

### 4.2 Ammonia Emissions

Table 2 presents the feedstock-specific mean and median NH_3_ emission factors from our literature survey. Boxplot visualizations of the NH_3_ data are provided in the Supporting Information (Figure S2). As is the case with GHG emissions, the highest average NH_3_ emission factor is associated with manure, followed by OFMSW. Composting yard waste emits the least NH_3_. As discussed earlier, NH_3_ is a product of microbial decomposition of proteins in the composted waste, and a fraction of that nitrogen will ultimately be emitted as N_2_O. Elevated NH_3_ and N_2_O emissions can simply indicate that a protein-rich feedstock is being decomposed through the nitrification and denitrification processes, although these emissions can also be sensitive to compost management methods.^60^

**Table 2:** Summary of NH_3_ and VOC Emission Factor Data for Composting Raw Materials and Digestate

	NH_3_ emission factors (kg NH_3_/kg of wet feedstock)			VOC emission factors (kg VOC/kg of wet feedstock)		
feedstock	mean	median	sample size	mean	median	sample size
manure	2.04 × 10^–3^	1.64 × 10^–3^	44	6.06 × 10^–5^	6.06 × 10^–5^	2
OFMSW	1.03 × 10^–3^	2.79 × 10^–4^	29	1.71 × 10^–3^	3.60 × 10^–4^	13
sludge	7.70 × 10^–4^	3.27 × 10^–4^	13	1.77 × 10^–4^	1.80 × 10^–4^	3
yard waste	8.91 × 10^–5^	2.50 × 10^–5^	5	5.23 × 10^–4^	4.62 × 10^–4^	4
digestate	5.50 × 10^–4^	6.22 × 10^–5^	25	1.16 × 10^–4^	3.72 × 10^–5^	11

According to Andraskar et al. (2021),^82^ maintaining aerobic conditions is imperative for controlling NH_3_ and other odorous emissions because many of these compounds are produced from anaerobic processes.^82^ Bulking agents can increase porosity to facilitate better aeration; Zhang et al. (2021) found that composting kitchen waste emitted 62% more NH_3_ than composting a mixture of 85% kitchen and 15% garden waste.^83^ Shao et al. (2014) observed a similar effect on NH_3_ emissions as the bulking agent-to-substrate ratio increased.^84^ In terms of operational methods, there appear to be trade-offs in NH_3_ emission rates when forced aeration is used to maintain aerobic conditions. Several studies have found that intermittent aeration at lower rates reduced NH_3_ emissions during swine manure composting.^53,60,81^ This is generally supported by other experimental studies that have observed increases in NH_3_ emissions when forced aeration increases.^49,53,60^ In addition to managing aeration, composters can also use microbial inoculation to control NH_3_ emissions.^82,85^ Chen et al. (2022) measured an ∼20% reduction in NH_3_ emissions when composting sewage sludge with a compound bacterial consortium relative to the control.^85^

Unlike for N_2_O, there is not consistent evidence to suggest whether treating waste with AD prior to composting increases or decreases NH_3_ emissions. A study by Smet et al. (1999) measured odors from OFMSW composting, AD of OFMSW, and digested OFMSW composting and found that AD pretreatment reduced NH_3_ emissions by 73%.^86^ Even when including emissions during AD, composting raw OFMSW still emitted 72% more NH_3_ than combined AD and composting. Maulini-Duran et al. (2013) observed a 98% decrease in NH_3_ emissions when comparing raw sludge composting to digested sludge composting; however, as noted earlier, this study did not include a proper experimental control, as the material came from two entirely different facilities.^54^ Rincón et al. (2019) compared emissions from 15 different feedstocks, including 5 different digestates, and found that on average and a wet mass basis, NH_3_ emissions were 87% lower for digested materials compared to raw feedstocks.^87^ Like Maulini-Duran et al., Rincón et al. did not include proper experimental controls since the feedstocks all came from different sites and the digestates were not derived from the same material in the raw feedstocks. In contrast, Colón et al. (2012) found that NH_3_ emissions from OFMSW composting roughly doubled with AD pretreatment.^77^ Similarly, Li et al. (2018) observed an increase of up to 40% in NH_3_ emissions from manure composting when materials first underwent AD.^63^ The mean NH_3_ emission factor for composting post-AD materials (including OFMSW, manure, and sludge) is lower than that for raw OFMSW or manure but on the same order of magnitude as the mean value for composting sludge (Table 2). Generally, composting untreated yard waste appears to emit less NH_3_ than composting digestates, but there is no available emissions data on composting digested yard waste.

### 4.3 VOC Emissions

Of all the compounds discussed in this review, VOCs are the least commonly reported, and although individual compounds may have differing effects on local odor concerns and air quality, VOC emissions are typically summed and reported as a total mass. VOCs include ketones, alcohols, terpenes, and other carbon compounds that can participate in atmospheric reactions with the exception of carbon monoxide, carbon dioxide, carbonic acid, metallic carbides or carbonates, and ammonium carbonate as defined by the EPA.^88^ CH_4_ is a VOC, although it is often reported separately because of its relevance for climate forcing; remaining VOCs are reported as non-methane VOCs. All of the values reported in this section and in Table 2 exclude CH_4_. The surveyed literature includes a total of 33 VOC emission factors, 11 of which are for digestate composting. The summary data broken down by feedstock type is provided in Table 2, but the sample sizes are limited (*n* < 5 for all raw feedstocks except OFMSW), so those emission factors should be used with caution. Further research is required to establish accurate distributions of these feedstock-specific emission factors. Without controlling for feedstock type, the mean emission factor is 8.14 × 10^–4^, and the median is 1.06 × 10^–3^ kg VOC per kg of wet feedstock for composting.^11,54,72,77,84,86,87,89,90^ If pressed to assume a nonzero value, researchers and practitioners may choose to use a median or mean value that excludes digestate. For nondigestate feedstocks (including sludge, OFMSW, and yard waste), the mean emission factor is 1.18 × 10^–3^, and the median is 2.1 × 10^–4^ kg VOC per kg of wet feedstock (*n* = 21).

All of the surveyed VOC emission factors for digestate composting are at least an order of magnitude lower than the average for nondigestate composting, suggesting AD may reduce VOC emissions from composting. Most of the surveyed literature provides evidence to support this conclusion with the exception of Colón et al. (2012) which measured higher VOC emissions from composting post-AD OFMSW compared to raw OFMSW.^77^ Smet et al. (1999) compared the VOC emissions from composting and AD of OFMSW, as they did with NH_3_, and the results show a 99% reduction in VOC emissions from composting when AD pretreatment was used. Expanding the system boundary, the combined AD and composting scenario had 63% fewer VOC emissions than direct OFMSW composting.^86^ Maulini-Duran et al. (2013) observed a decrease in VOC emissions from composting post-AD sludge relative to sludge that was not treated with AD.^54^ More recently, Rincón et al. (2019) compared odorous emissions from each raw feedstock type and digestate type with the exception of digested yard waste for which they had no data.^87^ On average, VOC emissions from digestate composting are 94% lower than those from raw material composting. When controlling for feedstock type, the VOC emission factors for composting digested materials are consistently lower than their raw counterparts, but as stated before, Rincón et al. did not include ideal experimental controls.^87^ Beyond AD, other technology options exist to specifically target and reduce VOC emissions from composting; these include but are not limited to pretreatment techniques, incineration, biotrickling filters, bioscrubber technology, and membrane bioreactors.^82,91^

## 5 Conclusions

Properly accounting for composting emissions, and for organic waste management-related emissions in general, in an LCA can be exceptionally challenging. There are still large gaps in the empirical data available for the range of materials that can be composted and the key greenhouse gases and air pollutants. More fundamentally, there is a limited scientific understanding of the complex microbial communities that break down plant matter, and emissions estimates are likely to evolve as scientists gain an improved understanding of the complex chemical and biological mechanisms at work in these environments. However, by analyzing data reported across the literature and disaggregating emission factors based on pile management strategies and starting material, basic patterns emerge that can inform best-estimates for use in future analyses.

Our findings suggest that N_2_O is typically the dominant contributor to the GWP_100_ of direct emissions from composting operations in properly aerated piles/windrows, assuming biogenic CO_2_ emissions do not have a net climate impact. When controlling for feedstock type, N_2_O accounts for 45–79% based on mean values and 59–91% based on median values of total GHG emissions on a GWP_100_ basis. Yard waste is a notable exception where GHG emissions are dominated by CH_4_ (80% of GWP_100_ based on mean values or 83% based on median values), likely because of its high C:N ratio compared to other waste types such as food waste and manure. Among observed feedstock types, N_2_O emissions appear to be highest for manure and lowest for yard waste and may be influenced by initial VS content. N_2_O emissions seem to be impacted by whether the incoming material was previously processed in an AD facility, and this paper outlines a suggested method for adjusting the estimated N_2_O emission factor based on VS reductions during AD. Conversely, CH_4_ appears to be primarily related to whether the pile or windrow is adequately aerated. The impact of VS reduction, through AD or otherwise, prior to composting did not appear to have a substantial impact on CH_4_ emissions, although direct comparisons in the empirical data are extremely limited and warrant further study. Assuming CH_4_ emissions are heavily influenced by pile management, reducing GHG emissions from well-managed, properly aerated compost piles may require more focus on the composition and quality of feedstock materials to reduce N_2_O emissions.

Regarding NH_3_ and VOCs, the available data suggests that treating waste with AD prior to composting may reduce these emissions, but more measurements are required to definitively support this conclusion. This uncertainty is echoed by inconsistent results and disagreement in current scientific literature, emphasizing the need for further research in this area. Reducing these emissions is a key part of improving air quality in local and surrounding communities not only because of odor concerns but also because both NH_3_ and VOC contribute to atmospheric formation of PM_2.5_, which has significant human health impacts. Therefore, though AD does not have a clear benefit with respect to limiting GHG emissions from composting, it can still play a role in effective organic waste management because of its potential to reduce other harmful emissions.

## Supporting Information Available

Relevance of ammonia emissions; detailed descriptions of measurement methods; carbon dioxide emissions from composting; boxplot distribution of carbon dioxide emission factors for composting, Figure S1; discussion of net emissions and offsets from compost application to soil; comparison of compost emissions to landfill emissions; and boxplot distribution of ammonia emission factors for composting, Figure S2 (PDF)
Table S1 including emission factors (XLSX)
