# Correction to “Greenhouse Gas and Air Pollutant Emissions from Composting”

**DOI:** 10.1021/acs.est.5c08656

**Published:** 2025-07-15

**Authors:** Sarah L. Nordahl, Chelsea V. Preble, Thomas W. Kirchstetter, Corinne D. Scown

Of 140 composting scenarios included in the originally published analysis, a single scenario involving yard waste and three corresponding GHG emission factors (CO_2_, CH_4_, and N_2_O) reflect data from Andersen et al. (2010). The emission factors from this study were rereported by Vergara and Silver (2019) and then used in this paper as reported by Vergara and Silver. However, Vergara and Silver (2019) incorrectly reported the units for the CH_4_ and N_2_O emission factors (2.4 g of CH_4_/kg and 0.06 g of N_2_O/kg). The correct emission factors from Andersen et al. (2010) are 2.4 kg of CH_4_-C/t and 0.06 kg of N_2_O-N/t, which correspond to 3.2 g of CH_4_/kg and 0.094 g of N_2_O/kg, respectively. The correct emission factors are on the same order of magnitude as the incorrect values. Rerunning the analysis with the corrected values increases the mean CH_4_ and N_2_O emission factors for yard waste by only 5% and 11%, respectively. The median values are unchanged. We also found that our value for emissions of N_2_O/kg of wet feedstock for composting of OFMSW had been inadvertently rounded to 6.8 × 10^–5^ in the original manuscript. We have updated the value 6.82 × 10^–5^. See the corrected version of Table 1 (changes shown in bold).

**Table 1:** Summary of GHG Emission Factor Data for Composting Raw Materials^a^

		emission factor				
		kg of pollutant/kg of wet feedstock		kg of CO_2e_/kg of wet feedstock		
feedstock	pollutant	mean	median	mean	median	sample size
manure	CH_4_	2.82 × 10^–3^	1.21 × 10^–3^	7.90 × 10^–2^	3.39 × 10^–2^	41
	N_2_O	3.54 × 10^–4^	1.62 × 10^–4^	1.05 × 10^–1^	4.83 × 10^–2^	45
	CO_2_	1.40 × 10^–1^	1.47 × 10^–1^	1.40 × 10^–1^	1.47 × 10^–1^	30
OFMSW	CH_4_	8.79 × 10^–4^	2.43 × 10^–4^	2.46 × 10^–2^	6.80 × 10^–3^	21
	N_2_O	**6.82 × 10^–5^**	7.50 × 10^–5^	2.03 × 10^–2^	2.24 × 10^–2^	19
	CO_2_	5.63 × 10^–2^	4.30 × 10^–2^	5.63 × 10^–2^	4.30 × 10^–2^	3
sludge	CH_4_	2.34 × 10^–4^	4.50 × 10^–5^	6.55 × 10^–3^	1.26 × 10^–3^	7
	N_2_O	8.36 × 10^–5^	4.36 × 10^–5^	2.49 × 10^–2^	1.30 × 10^–2^	7
	CO_2_	1.75 × 10^–2^	1.75 × 10^–2^	1.75 × 10^–2^	1.75 × 10^–2^	2
yard waste	CH_4_	**2.17 × 10^–3^**	1.23 × 10^–3^	**6.08 × 10^–2^**	3.44 × 10^–2^	7
	N_2_O	**5.03 × 10^–5^**	2.27 × 10^–5^	**1.50 × 10^–2^**	6.76 × 10^–3^	7
	CO_2_	1.71 × 10^–1^	1.56 × 10^–1^	1.71 × 10^–1^	1.56 × 10^–1^	4

a Digestate is excluded from this table because of variation in the original raw feedstock materials.

Additionally, the originally published paper included a typo in Table 2. The mean NH_3_ emission factor for OFMSW is 1.3 × 10^–3^, not 1.03 × 10^–3^. See corrected version below (changes shown in bold).

**Table 2:** Summary of NH_3_ and VOC Emission Factor Data for Composting Raw Materials and Digestate

	NH3 Emission Factors (kg NH3/kg of wet feedstock)			VOC Emission Factor (kg VOC/kg of wet feedstock)		
feedstock	mean	median	sample size	mean	median	sample size
manure	2.04 × 10^–3^	1.64 × 10^–3^	44	6.06 × 10^–5^	6.06 × 10^–5^	2
OFMSW	**1.30 × 10^–3^**	2.79 × 10^–4^	29	1.71 × 10^–3^	3.60 × 10^–4^	13
sludge	7.70 × 10^–4^	3.27 × 10^–4^	13	1.77 × 10^–4^	1.80 × 10^–4^	3
yard waste	8.91 × 10^–5^	2.50 × 10^–5^	5	5.23 × 10^–4^	4.62 × 10^–4^	4
digestate	5.50 × 10^–4^	6.22 × 10^–5^	25	1.16 × 10^–4^	3.72 × 10^–5^	11

These errors do not affect the conclusions of the report.

