# Supplementary material for: Greenhouse Gas and Air Pollutant Emissions from Composting
Source: Environ Sci Technol. 2023 Jan 31;57(6):2235–47. doi: 10.1021/acs.est.2c05846 (PMC9933540; doi:10.1021/acs.est.2c05846)
Supplement: Supplementary file 1 — es2c05846_si_001.pdf [file es2c05846_si_001.pdf]

Supporting Information

Greenhouse gas and air pollutant emissions from composting

Sarah L. Nordahl[1,2], Chelsea V. Preble[1,2], Thomas W. Kirchstetter[1,2], Corinne D. Scown[1,3,4,5*]

[1]Energy Technologies Area, Lawrence Berkeley National Laboratory, 1 Cyclotron Road, Berkeley, CA 94720

[2]Department of Civil and Environmental Engineering, University of California, Berkeley, Berkeley, CA 94720

[3]Biosciences Area, Lawrence Berkeley National Laboratory, 1 Cyclotron Road, Berkeley, CA 94720

[4]Joint BioEnergy Institute, 5885 Hollis Street, Emeryville, CA 94608

[5]Energy & Biosciences Institute, University of California, Berkeley, Berkeley, CA 94720

Number of Pages: 15
Number of Figures: 2
Number of Tables: 1 (Table S1 provided in separate Excel file)

1 Relevance of Ammonia Emissions

In addition to being malodorous, NH_3 emissions are an important precursor to $PM_{2.5}$ formation, so it is not uncommon for studies to report NH_3 emissions alongside GHGs. Once in the atmosphere, NH_3 can react with nitric acid (HNO_3) to form particle-phase ammonium nitrate (NH_4NO_3) and/or sulfuric acid (H_2SO_4) to form particle-phase ammonium sulfate ($(NH_4)_2SO_4$). The precursors that NH_3 reacts with, H_2SO_4 and HNO_3, are formed in the atmosphere as a result of NO_x and SO_x emissions from power plants, motor vehicles, and other combustion activities. Alternatively, NH_3 may undergo wet or dry deposition, accumulating in nearby water bodies or on soil surfaces, where a portion of the nitrogen is later converted to N_2O.[1] Predicting the impact of NH_3 emissions on $PM_{2.5}$ concentrations has proved challenging and integrated assessment models vary in their predictions.[2] Nonetheless, Tschofen et al. (2019) found that NH_3 is responsible for the largest share of air quality-related monetized health damages from the agriculture sector.[3] A prior study found that NH_3 dominated the total social costs—including both climate change impacts and air quality-related human health damages—in any organic waste processing scenario that included composting.[4]

2 Measurement Methods

1.1 Flux Chambers

One commonly used approach to quantify composting emissions is to place static, open-bottomed chambers with small surface area footprints on the emitting surface. There are two common variants of these flux chambers, one in which swept air flow from over the emitting surface is sampled and a modified version that relies on diffusive transport to accumulate emitted gas into a headspace volume that is then sampled. In the first, ultrapure or "zero" air that is free of the air pollutants of interest is introduced at the inlet and gas samples are taken from the chamber outlet flow, either collected into a canister or bag for laboratory analysis or measured in situ.[5–7] This method follows the U.S EPA protocol for measuring gaseous emission rates from land surfaces.[8] A modified approach for non-aerated windrows where diffusion is the main emission mechanism outside of pile turning periods is to use closed/airtight or vented chambers, where the concentrations measured in the chamber headspace can be related to gas fluxes.[9–15] With flux chambers, the small surface area may not be representative of the entire emitting surface and measurements can be temporally constrained in resolution. The chamber can also introduce pressure and concentration gradients that impact emission fluxes from the windrow surface.

1.2 Gas Probes

To collect cross-sectional gas samples from within the pore space of the composting pile rather than the emitting surface, probes are inserted into the windrow at varying depths.[9,11,13,15,16] The probes are flushed prior to collecting a sample to ensure that gas from the pore space itself is sampled rather than the probe's dead volume. From these measurements, it is possible to determine the spatial distributions of gas concentrations within the windrow, providing insight into composting dynamics like pockets of anaerobic activity with elevated CH_4 concentrations.

1.3 Wind Tunnels

Wind tunnels have been used as an alternative to flux chambers when the high water content of sampled gas has been an issue.[7] These static, flow-through enclosures also have open bottoms and are inserted ~1 cm into the windrow surface. A fan introduces ambient air dilution to emitted gas, which is then collected into canisters or bags. These tunnels cover larger surface areas than flux chambers, the dilution offers more control of water content in samples, and the greater air exchange rate is more similar to ambient conditions. Similar to flux chambers, though, the small surface area sampled may not be representative of the entire windrow and measurements can be temporally constrained. The inlet and outlet air must also be simultaneously sampled to properly calculate emission flux, as ambient air with non-zero concentrations of the air pollutants of interest is used for dilution rather than zero air.

1.4 Open Emission Chambers

To capture emissions from across the emitting surface, open chambers are built over the windrow, with air flowing in and out of the control volume either naturally or by a ventilation system.[17–21] The difference in measured concentrations in the incoming and outgoing air can be used to calculate the emissions inside the chamber from the composting pile. In theory, this sampling approach does not alter the conditions inside the chamber, and compost can be maintained in the usual manner, for example aeration with pile turning. If pollutant analyzers with a fast time response are used, this measurement can be temporally-resolved and show emissions changes over time. However, as it integrates emissions over the entire windrow, it will not discern spatial variability in emissions across the surface.

1.5 Tracer Releases

If an inert gas is released at the emission source at a known rate, the downwind ratio of tracer gas to pollutant concentration can be measured to determine the pollutant emission rate.[13,22] These measurements are relatively simple to conduct, but the emission point may not be representative of the entire composting surface. It may also be difficult to isolate a specific windrow from facility-wide emissions, if there are multiple emission sources of the pollutant species of interest.

1.6 Inverse Dispersion Analysis

For sources with known geometry, emission rates can be determined with a dispersion model that pairs measured downwind concentrations and local meteorology.[22] This micrometeorological analysis technique gives an integrated measure of emission flux, but may also be limited to facility-wide emission rates if windrows are in close proximity to each other or if there are other nearby emission sources of the pollutant of interest. While the measurements are relatively simple to conduct, the analysis relies on an accurate dispersion model.

1.7 Micrometeorological Mass Balance

Another micrometeorological approach relies on mass balance, in which pollutant fluxes in and out of a control volume surrounding the emission source are determined from measured gas concentrations and wind dynamics.[23] This method captures the integrated emission rate from an isolated windrow or full-scale facility operations, and measurements can be made across the composting cycle to characterize emission rates as a function of time.

1.8 High-Density Spot Sampling

At some facilities, the density of windrow placement and local environmental conditions preclude the use of open emission chambers or micrometeorological approaches to capture the emissions from individual composting windrows. Moreover, when spatial heterogeneity across the emitting surface is expected—such as with heterogeneous OFMSW or digestate feedstocks compared to more uniform materials like yard waste—small surface area footprint approaches like flux chambers and gas probes may not be sufficient to capture a representative sample of emissions. In these cases, a high-density spot sampling method can be employed instead.[24] With this technique, the characteristic emission rate for individual windrows is calculated from the measured forced aeration flow and numerous spot gas samples that are collected into bags from across the composting surface and later analyzed in the laboratory. Multiple windrows can be sampled in a given day to compare emission rates across the composting cycle. This approach is intensive before and after sampling, though, in terms of sample bag preparation and analysis. Measurements are intermittent across the composting cycle rather than continuous, producing snapshots of emissions at given points of time. This method is also limited to force-aerated windrows and cannot be applied to composting piles that are turned or naturally/passively aerated.

1.9 Tradeoffs Among Common Measurement Methods

When selecting composting studies to draw from for use in a broader environmental analysis, one could reasonably ask whether particular measurement methods are superior and should be given priority. There is a clear tradeoff among the methods discussed here between specificity to the feedstock of interest and the degree to which the measurements accurately represent

emissions in commercial composting conditions. In most cases, it is not realistic to run experiments in which large-scale windrows are composed of a single material, nor would a mixed windrow result in emissions equal to the sum of its parts in isolation. Generally, measurements taken in the field from actual composting operations are preferable to lab-based studies. However, lab-based measurements can be valuable, particularly when done in combination with field studies, by better characterizing the relative impact of specific changes to feedstock composition or composting conditions (e.g., moisture content, pH, etc.) on emissions.

Of the field-based measurement methods, there is no single approach that is obviously superior. Spot sampling methods, which include flux chambers, gas probes, wind tunnels, tracer releases, and high-density spot sampling, can provide some spatial resolution of emissions from a composting windrow. Sampling size and distribution across the windrow are important factors in calculating a total, cumulative emission factor from these methods. Therefore, one should be wary of emission factors from studies that employed one of these methods with a small number of samples or if the spatial distribution of sampling locations along the windrow or pile is limited. In addition to spatial distribution, the temporal distribution of measurements over the composting cycle is equally important. Commercial composting takes 3–6 months, and emissions will vary across the mesophilic, thermophilic, and maturation phases. If a study uses flux chambers, wind tunnels, tracer releases or high-density spot sampling, it is important that measurements were taken with some regularity over the entire composting cycle to determine a final emission factor.

Employing spot sampling measurements at multiple locations across a windrow for an entire multi-month composting cycle is labor-intensive and may not be practical in some cases. Other approaches, like open emission chambers, inverse dispersion analysis, and micrometeorological mass balances, can offer windrow-wide or facility-wide results but do not offer spatially resolved results. The downside to such approaches is that emissions cannot be easily connected back to the types of material being composted, the composting conditions at a large facility that accepts a range of wastes, or the composting dynamics within the windrow itself (e.g., nonuniform aeration that leads to pockets of anaerobic activity). Moreover, some of these results can be restricted by the detection and quantification limits of the pollutant analyzers used, such that emissions may be non-zero but not detectable downwind of the source. Ultimately, researchers and other practitioners may choose to draw emission factors from multiple studies and use a range or probability distribution when incorporating composting emissions into life-cycle assessments and other environmental impact studies. However, the information provided here may be useful in selecting the most rigorous and representative studies for a given application.

1.10 Impact of Measurement Methods

Our analysis of the impact of measurement methods was inconclusive because there are not enough comparable studies to find meaningful results. The data collected for this systematic

review does not provide sufficient evidence to suggest that any particular method consistently overestimates or underestimates emission measurements more than other methods. Researchers and practitioners should select emission factors based on their suitability for the specific analysis in which they are being used, and the quality of the associated study's measurement approach (e.g., sufficient temporal and spatial distribution of sampling in spot sample approaches). More research observing the same feedstocks and composting conditions with varied measurement techniques is required to better understand how these methods may skew results.

3 Carbon Dioxide Emissions from Composting

Figure S1 shows the results for biogenic CO_2, excluding one major outlier from [16], which observed an unusually high emission factor of 0.87 kg CO_2 per kg of green waste composted. During composting, a fraction of initial carbon in the feedstock is emitted, primarily as CO_2 and CH_4, and the remaining carbon is retained in the material and can contribute to soil organic carbon once applied to land.[23,25] If a composting operation is not well aerated, microbes will consume the organic material more slowly, leading to elevated CH_4 emissions and a reduction in CO_2 emissions. This negative correlation between CO_2 and CH_4 emissions has been measured by Jiang et al. (2011) and Chowdhury et al. (2014).[26,27] This finding is not consistent across the literature, however, with other studies reporting a positive correlation between CO_2 and CH_4 emissions.[19,20,24,28] Many of these studies provide limited data, with samples ranging from just 3 to 9 measurement pairs.[28,26] Therefore, we cannot recommend using measured CO_2 as a good predictor of the relative magnitude of CH_4 emissions (or any other pollutants) based on the data available.

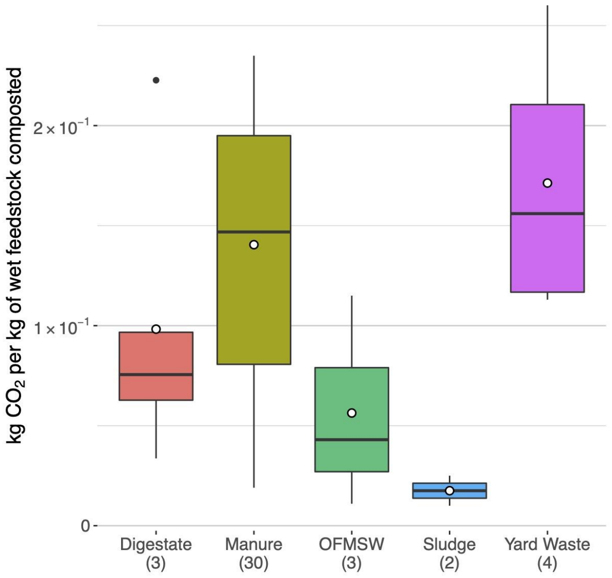

Figure S1. Distributions of CO_2 emission factors for composting reported in the literature. The sample size (n) of data points contributing to each boxplot is indicated in the x-axis labels. The mean values for the boxplot data are indicated by the open point symbols, while outliers are shown as closed circles.

4 GHG Emissions Implications of Landfilling and Land Application

4.1 Net Emissions and Offsets From Compost Application to Soils

Composting produces nutrient-rich material that can be beneficial to plant growth, contribute to pest and disease prevention, and offset the need for synthetic mineral fertilizers.[29] The benefits of compost application to soils include increased soil organic matter, stability, and water retention.[30] For this to hold true, the compost must be applied in an agricultural application, although some benefits may still be achieved in landscaping applications. Compost must also be applied at the agronomic rate, meaning the recommended application rate to achieve optimum plant growth. Assuming that all material sent to commercial composting operations will ultimately be used beneficially as compost in agricultural applications is likely too optimistic, and the market for compost will vary regionally. For example, a 2017 market analysis conducted by CalRecycle in California (U.S.) indicated that 22% of output from in-state composters went to landfills, mostly as alternative daily cover.[31] Researchers quantifying the life-cycle GHG benefits of waste diversion must be mindful of the fact that, if supply of finished compost exceeds demand, the marginal use-phase benefits of compost may be diminished or near-zero if it is either used for alternative daily cover or applied at levels exceeding the agronomic rate. An adjustment factor may be necessary to account for the fraction of finished compost not being used in an agronomic application.

If compost is applied to agricultural soils, there are two key drivers of net emissions/offsets: (1) increasing crop yields and thus reducing land and other inputs needed to produce the same amount of agricultural product; and (2) reducing the need for mineral fertilizer production and application. Pest prevention and associated reductions in pesticide use are a third contributor to offsets, but these impacts are complex, highly variable, and too ambiguous to be useful from an LCA perspective.[29]

Compost use as a soil amendment allows for the slow release of nitrogen on the scale of multiple years, as nitrogen in the compost is immobilized when it is taken up by microbes during the composting process and is only released as those microbes die and their cell walls lyse.[32] For instance, Sullivan et al. (2003) observed a continued increase in soil organic matter and crop yields for seven years after a one-time application of compost.[33] Sullivan et al. (2003) also found

that composting feedstocks with higher nitrogen content, such as food waste, produced a soil amendment that resulted in greater crop yield benefits.

Offsetting nitrogen fertilizer use is particularly important with respect to net GHG emissions because fertilizer production (e.g., urea) is energy- and emissions-intensive to produce.[34] Compost is most commonly used in addition to fertilizers, as opposed to a full replacement.[35] Given the complementary nature of synthetic fertilizers with compost when used concurrently to increase agricultural yields, there may not be a one-to-one substitution between the two when adjusted based on total available nitrogen. This is further complicated by the lack of consistency in compost quality and composition, particularly for composted OFMSW.[36] Favoino and Hogg (2008) estimate that a one-time application of 10 tonnes of compost has the potential to displace 190 kg of nitrogen and save from 160 kWh up to nearly 1600 kWh of energy, although it is unclear if this refers to primary or secondary energy.[34] Although the exact value of synthetic nitrogenous fertilizer use that can be offset through compost application is uncertain, there does appear to be broad consensus that fertilizer application, and thus upstream emissions from its production, can be reduced through compost application to agricultural land. Application of nitrogenous fertilizer also contributes to GHG emissions after it is applied through N_2O fluxes to the atmosphere. The extent to which compost application can reduce these fluxes, if at all, is uncertain and dependent on local conditions, the existing soil biological community, fertilizer characteristics, compost characteristics, and management practices.[34,37] For example, Ryals and Silver (2013) did not observe significant changes to soil CH_4 or N_2O fluxes after compost application.[38] In the absence of consistent empirical evidence that suggests otherwise, the most defensible assumption in life-cycle assessment models may be that compost application does not positively or negatively impact CH_4 or N_2O fluxes from agricultural soils.

The degree to which compost application results in greater carbon sequestration or residence time in soils is highly uncertain. Soil organic matter, which generally refers to the organic fraction of soil excluding undecayed animal and plant matter, is particularly important to crop growth because it directly impacts nitrogen availability, soil water retention, and other physical soil properties.[35,39] However, the long-term stability of that material remains an open question; the soil science community began transitioning away from the concept of humus as stable and resistant to decomposition.[40] Researchers should exercise caution in using soil carbon sequestration factors for compost, particularly if the data is sourced from literature published prior to the recent shift in scientists' understanding of what kinds of molecules can be metabolized by the soil microbial community. Studies do suggest that when compost is applied to grasslands, the increase in net primary productivity results in an increase in above- and below-ground carbon stocks.[34,41] For example, in the analysis of compost application to California grasslands presented in Breunig et al. (2019), a one-time application of compost was assumed to provide an additional 0–4.7 tonnes of soil carbon per hectare of amended land and the increase in soil carbon was estimated to last 30 years.[41] The question of how this accumulated carbon

remains in soils remains largely unresolved because of limits in the scientific community's understanding of soil microbial communities. Even the lignin fraction of plants, which was long thought to limit decomposition of plant litter, can degrade more quickly than other components of plants under favorable conditions.[42] For this reason, it is challenging to recommend an approach to accounting for soil organic carbon impacts associated with compost application. As with CH_4 or N_2O fluxes, the most defensible assumption in the near-term may be that compost application does not result in any net accumulation of carbon in soils.

4.2 Comparing Composting Emissions to Landfill Emissions

The most common alternatives to composting depend on both the type of material in need of management (e.g., OFMSW, manure, yard waste, digestate) and the country in which it is being managed. For example, the U.S. landfills most of its OFMSW while manure may be left on land or stored in open lagoons.[43,44] For OFMSW, there is a clear consensus among the majority of life-cycle emissions studies that composting organic waste results in lower net GHG emissions relative to landfilling.[4,45] There is far less consensus on the actual emissions footprint of landfilling different types of organic wastes.

The GHG footprint of landfills is dominated by fugitive CH_4 emissions, even for those with gas capture systems in place, and there are many different strategies for measuring these emissions.[46,47] Even with accurate measurements, it is difficult to draw a causal link between specific types and quantities of waste sent to landfills and the resulting emitted CH_4. For this reason, commonly used emission factors are based on lab experiments, in which anaerobic decomposition of specific types of materials is stimulated to determine decay rates and CH_4 generation, and those values are later adjusted to account for landfill gas capture rates.

One of the most frequently cited sources for GHG emission factors for landfilling is the U.S. Environmental Protection Agency's Waste Reduction Model (WARM),[48] which is based on experimental work by Barlaz (1998) and De la Cruz and Barlaz (2010).[49,50] However, these experiments simulated enhanced landfilling, where decomposition is purposefully accelerated. Barlaz (1998) measured methane emissions with the decay rate of material under optimal conditions in the laboratory. Organic materials were shredded, seeded, and incubated at 40 ºC, and were supplied with phosphate and ammonia to ensure that degradation was not nutrient-limited. Leachate (i.e., residual liquid) was also neutralized and subsequently recycled to the reactor to ensure that the pH did not slow microbial activity. These experimental decay rates represent an upper bound rather than what would occur in a typical industrial scale landfill, where waste does not routinely undergo shredding, nor are other measures taken to neutralize the pH or supply nutrients to speed up degradation. In a similar example, De la Cruz and Barlaz (2010) estimated feedstock specific decay rates using data from Eleazer et al. (1997),[51] which simulated enhanced landfilling like Barlaz (1998). Realistic decay rates are important for estimating fugitive emissions because, in landfills with gas capture systems, most fugitive CH_4

emissions occur in the time between when waste is placed in the landfill and the individual cell is capped. Faster decay rates result in more fugitive CH_4 emissions.

It is possible that reliance on measured values from enhanced landfilling experiments has resulted in systematic overestimation of CH_4 emissions and the extent of material degradation in landfills. For instance, based on Barlaz (1998), WARM uses an emission factor of 6.38×10^{-2} kg of CH_4 per wet kg of food waste landfilled, in contrast to the emission factor of 1.30×10^{-2} kg of CH_4 per wet kg of food waste as measured by Behera et al. (2010).[52] In the Behera et al. study, food waste leachate sourced from a food waste recovery plant was filtered through a sieve and directly fed into an anaerobic reactor in a lab. Food waste leachate, which is a dense liquid, is a suitable proxy for raw food waste, since it makes up 70–90% of the food waste material collected and stored at food waste recovery plants. The measured emission factor from this experiment was about 80% lower than that estimated by Barlaz. Because of the overestimation associated with enhanced landfilling in the Barlaz study, the reduced emission factor from Behera et al. (2010) was preferred for use in Nordahl et al. (2020).[4] Even when considering the reduced landfill emission factor, composting still presents as the favorable option for handling OFMSW, with a per-tonne CH_4 emission factor (8.79×10^{-4} kg of CH_4 per wet kg of OFMSW, Table 2) that is two orders of magnitude lower than that assumed by WARM or found in Behera et al. (2010) for landfilling.

5 Ammonia Emissions

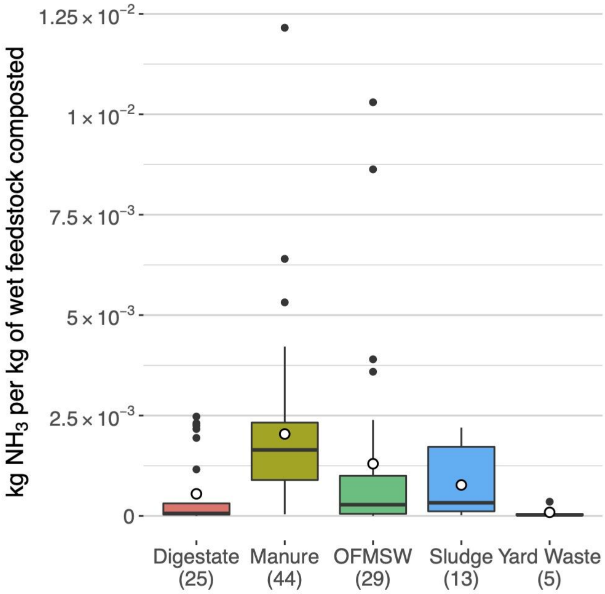

Figure S2. Distributions of NH_3 emission factors for composting reported in the literature. The sample size (n) of data points contributing to each boxplot is indicated in the x-axis labels. The mean values for the boxplot data is indicated by the open point symbols, while outliers are shown as closed circles.

References

(1) U.S. EPA. *Inventory of U.S. Greenhouse Gas Emissions and Sinks 1990-2018*; EPA 430-R-20–002; United States Environmental Protection Agency, 2019.

(2) Gilmore, E. A.; Heo, J.; Muller, N. Z.; Tessum, C. W.; Hill, J. D.; Marshall, J. D.; Adams, P. J. An inter-comparison of the social costs of air quality from reduced-complexity models. *Environmental Research Letters* **2019**, *14*, 074016.

(3) Tschofen, P.; Azevedo, I. L.; Muller, N. Z. Fine particulate matter damages and value added in the US economy. *Proc Natl Acad Sci USA* **2019**, *116*, 19857–19862.

(4) Nordahl, S. L.; Devkota, J. P.; Amirebrahimi, J.; Smith, S. J.; Breunig, H. M.; Preble, C. V.; Satchwell, A. J.; Jin, L.; Brown, N. J.; Kirchstetter, T. W.; Scown, C. D. Life-cycle greenhouse gas emissions and human health trade-offs of organic waste management strategies. *Environ. Sci. Technol.* **2020**, *54*, 9200–9209.

(5) Büyüksönmez, F. Full-scale VOC emissions from green and food waste windrow composting. *Compost Sci. Util.* **2012**, *20*, 57–62.

(6) Zhu-Barker, X.; Bailey, S. K.; Paw U, K. T.; Burger, M.; Horwath, W. R. Greenhouse gas emissions from green waste composting windrow. *Waste Manag.* **2017**, *59*, 70–79.

(7) Kumar, A.; Alaimo, C. P.; Horowitz, R.; Mitloehner, F. M.; Kleeman, M. J.; Green, P. G. Volatile organic compound emissions from green waste composting: Characterization and ozone formation. *Atmos. Environ.* **2011**, *45*, 1841–1848.

(8) EPA. *Measurement of gaseous emission rates from land surfaces using an emission isolation flux chamber*; EPA/600/8-86/008; U.S. EPA, 1986.

(9) Czepiel, P.; Douglas, E.; Harriss, R.; Crill, P. Measurements of N_2O from composted organic wastes. *Environ. Sci. Technol.* **1996**, *30*, 2519–2525.

(10) Hellmann, B.; Zelles, L.; Palojarvi, A.; Bai, Q. Emission of climate-relevant trace gases and succession of microbial communities during open-windrow composting. *Appl. Environ. Microbiol.* **1997**, *63*, 1011–1018.

(11) Beck-Friis, B.; Pell, M.; Sonesson, U.; Jönsson, H.; Kirchmann, H. Formation and emission of N_2O and CH_4 from compost heaps of organic household waste. *Environmental Monitoring and Assessment* **2000**, *62*, 317–331.

(12) Hao, X.; Chang, C.; Larney, F. J. Carbon, nitrogen balances and greenhouse gas emission during cattle feedlot manure composting. *J. Environ. Qual.* **2004**, *33*, 37–44.

(13) Andersen, J. K.; Boldrin, A.; Samuelsson, J.; Christensen, T. H.; Scheutz, C. Quantification of greenhouse gas emissions from windrow composting of garden waste. *J. Environ. Qual.* **2010**, *39*, 713–724.

(14) Colón, J.; Cadena, E.; Pognani, M.; Barrena, R.; Sánchez, A.; Font, X.; Artola, A. Determination of the energy and environmental burdens associated with the biological treatment of source-separated Municipal Solid Wastes. *Energy Environ. Sci.* **2012**, *5*, 5731–5741.

(15) Chen, R.; Wang, Y.; Wang, W.; Wei, S.; Jing, Z.; Lin, X. N_2O emissions and nitrogen

transformation during windrow composting of dairy manure. *J. Environ. Manage.* **2015**, *160*, 121–127.
(16) Hellebrand, H. J. Emission of nitrous oxide and other trace gases during composting of grass and green waste. *Journal of Agricultural Engineering Research* **1998**, 265–275.
(17) Amon; Amon, Th.; Boxberger; Alt, Ch. Emissions of NH_3, N_2O and CH_4 from dairy cows housed in a farmyard manure tying stall (housing, manure storage, manure spreading). *Nutrient Cycling in Agroecosystems* **2001**.
(18) Fukumoto, Y.; Osada, T.; Hanajima, D.; Haga, K. Patterns and quantities of NH3, N2O and CH4 emissions during swine manure composting without forced aeration—effect of compost pile scale. *Bioresour. Technol.* **2003**, *89*, 109–114.
(19) Paillat, J.-M.; Robin, P.; Hassouna, M.; Leterme, P. Predicting ammonia and carbon dioxide emissions from carbon and nitrogen biodegradability during animal waste composting. *Atmos. Environ.* **2005**, *39*, 6833–6842.
(20) Amlinger, F.; Peyr, S.; Cuhls, C. Greenhouse gas emissions from composting and mechanical biological treatment. *Waste Manag. Res.* **2008**, *26*, 47–60.
(21) Maeda, K.; Hanajima, D.; Morioka, R.; Toyoda, S.; Yoshida, N.; Osada, T. Mitigation of greenhouse gas emission from the cattle manure composting process by use of a bulking agent. *Soil Sci. Plant Nutr.* **2013**, *59*, 96–106.
(22) Hrad, M.; Binner, E.; Piringer, M.; Huber-Humer, M. Quantification of methane emissions from full-scale open windrow composting of biowaste using an inverse dispersion technique. *Waste Manag.* **2014**, *34*, 2445–2453.
(23) Vergara, S. E.; Silver, W. L. Greenhouse gas emissions from windrow composting of organic wastes: Patterns and emissions factors. *Environmental Research Letters* **2019**, *14*, 124027.
(24) Preble, C. V.; Chen, S. S.; Hotchi, T.; Sohn, M. D.; Maddalena, R. L.; Russell, M. L.; Brown, N. J.; Scown, C. D.; Kirchstetter, T. W. Air pollutant emission rates for dry anaerobic digestion and composting of organic municipal solid waste. *Environ. Sci. Technol.* **2020**, *54*, 16097–16107.
(25) Brown, S.; Kruger, C.; Subler, S. Greenhouse gas balance for composting operations. *J. Environ. Qual.* **2008**, *37*, 1396–1410.
(26) Jiang, T.; Schuchardt, F.; Li, G.; Guo, R.; Zhao, Y. Effect of C/N ratio, aeration rate and moisture content on ammonia and greenhouse gas emission during the composting. *Journal of Environmental Sciences* **2011**, *23*, 1754–1760.
(27) Chowdhury, M. A.; de Neergaard, A.; Jensen, L. S. Potential of aeration flow rate and bio-char addition to reduce greenhouse gas and ammonia emissions during manure composting. *Chemosphere* **2014**, *97*, 16–25.
(28) Sun, X.; Lu, P.; Jiang, T.; Schuchardt, F.; Li, G. Influence of bulking agents on CH_4, N_2O, and NH_3 emissions during rapid composting of pig manure from the Chinese Ganqinfen system. *J. Zhejiang Univ. Sci. B* **2014**, *15*, 353–364.
(29) Unable to find information for 6489453.

(30) Boldrin, A.; Andersen, J. K.; Møller, J.; Christensen, T. H.; Favoino, E. Composting and compost utilization: accounting of greenhouse gases and global warming contributions. *Waste Manag. Res.* **2009**, *27*, 800–812.
(31) CalRecycle. *SB 1383 Infrastructure and Market Analysis*; California Department of Resources Recycling and Recovery, 2019.
(32) Al-Bataina, B. B.; Young, T. M.; Ranieri, E. Effects of compost age on the release of nutrients. *International Soil and Water Conservation Research* **2016**, *4*, 230–236.
(33) Sullivan, D. M.; Bary, A. I.; Nartea, T. J.; Myrhe, E. A.; Cogger, C. G.; Fransen, S. C. Nitrogen availability seven years after a high-rate food waste compost application. *Compost Sci. Util.* **2003**, *11*, 265–275.
(34) Favoino, E.; Hogg, D. The potential role of compost in reducing greenhouse gases. *Waste Manag. Res.* **2008**, *26*, 61–69.
(35) Weindorf, D. C.; Muir, J. P.; Landeros-Sánchez, C. Organic compost and manufactured fertilizers: economics and ecology. In *Integrating Agriculture, Conservation and Ecotourism: Examples from the Field*; Campbell, W. B.; Lopez Ortiz, S., Eds.; Issues in agroecology – present status and future prospectus; Springer Netherlands: Dordrecht, 2011; Vol. 1, pp. 27–53.
(36) Eggerth, L. L.; Diaz, L. F.; Chang, M. T. F.; Iseppi, L. Chapter 12 Marketing of composts. In *Compost science and technology*; Waste Management Series; Elsevier, 2007; Vol. 8, pp. 325–355.
(37) Dambreville, C.; Hallet, S.; Nguyen, C.; Morvan, T.; Germon, J.-C.; Philippot, L. Structure and activity of the denitrifying community in a maize-cropped field fertilized with composted pig manure or ammonium nitrate. *FEMS Microbiol. Ecol.* **2006**, *56*, 119–131.
(38) Ryals, R.; Silver, W. L. Effects of organic matter amendments on net primary productivity and greenhouse gas emissions in annual grasslands. *Ecol. Appl.* **2013**, *23*, 46–59.
(39) Johnston, A. E. Soil organic matter, effects on soils and crops. *Soil Use & Management* **1986**, *2*, 97–105.
(40) Lehmann, J.; Kleber, M. The contentious nature of soil organic matter. *Nature* **2015**, *528*, 60–68.
(41) Breunig, H. M.; Amirebrahimi, J.; Smith, S.; Scown, C. D. Role of digestate and biochar in carbon-negative bioenergy. *Environ. Sci. Technol.* **2019**, *53*, 12989–12998.
(42) Hall, S. J.; Huang, W.; Timokhin, V. I.; Hammel, K. E. Lignin lags, leads, or limits the decomposition of litter and soil organic carbon. *Ecology* **2020**, *101*, e03113.
(43) Niles, M. T.; Wiltshire, S. Tradeoffs in US dairy manure greenhouse gas emissions, productivity, climate, and manure management strategies. *Environ. Res. Commun.* **2019**, *1*, 075003.
(44) EPA. *Inventory of U.S. Greenhouse Gas Emissions and Sinks: 1990-2020 – Annex 3 Part B*; U.S. Environmental Protection Agency, 2022.

(45) Lou, X. F.; Nair, J. The impact of landfilling and composting on greenhouse gas emissions--a review. *Bioresour. Technol.* **2009**, *100*, 3792–3798.
(46) Mønster, J.; Kjeldsen, P.; Scheutz, C. Methodologies for measuring fugitive methane emissions from landfills - A review. *Waste Manag.* **2019**, *87*, 835–859.
(47) Duren, R. M.; Thorpe, A. K.; Foster, K. T.; Rafiq, T.; Hopkins, F. M.; Yadav, V.; Bue, B. D.; Thompson, D. R.; Conley, S.; Colombi, N. K.; Frankenberg, C. California's methane super-emitters. *Nature* **2019**, *575*, 180–184.
(48) EPA. Waste Reduction Model (WARM). **2018**.
(49) Barlaz, M. A. Carbon storage during biodegradation of municipal solid waste components in laboratory-scale landfills. *Global Biogeochem. Cycles* **1998**, *12*, 373–380.
(50) De la Cruz, F. B.; Barlaz, M. A. Estimation of waste component-specific landfill decay rates using laboratory-scale decomposition data. *Environ. Sci. Technol.* **2010**, *44*, 4722–4728.
(51) Eleazer, W. E.; Odle, W. S.; Wang, Y.-S.; Barlaz, M. A. Biodegradability of Municipal Solid Waste Components in Laboratory-Scale Landfills. *Environ. Sci. Technol.* **1997**, *31*, 911–917.
(52) Behera, S. K.; Park, J. M.; Kim, K. H.; Park, H.-S. Methane production from food waste leachate in laboratory-scale simulated landfill. *Waste Manag.* **2010**, *30*, 1502–1508.
